# Treatment of Cutaneous Squamous Cell Carcinoma with Immune Checkpoint Inhibitors in Special Populations

**DOI:** 10.5826/dpc.11S2a170S

**Published:** 2021-11-01

**Authors:** Paolo Bossi, Luigi Lorini

**Affiliations:** 1 Medical Oncology Unit, Department of Medical and Surgical Specialties, Radiological Sciences and Public Health, ASST Spedali Civili of Brescia, University of Brescia, Brescia, Italy.

**Keywords:** cutaneous squamous cell carcinoma, special population, auto-immune disease, immunosuppression, immunodepression

## Abstract

Cutaneous squamous cell carcinoma (cSCC) may develop in patients with dysregulated immune activation (pre-existing autoimmune diseases or immunosuppression due to hematopoietic/solid organ transplant recipients), patients with a compromised immune function (long-term immunosuppression), and patients carrying chronic viral infections, or those affected by lymphoproliferative diseases. It should be also considered that patients presenting with immunosuppression have a high incidence of cSCC (65–250-times higher than general population), highlighting the central role played by the immune system in the development of cSCC. All these cases must be considered as “special populations” for treatment with immune checkpoint inhibitors (ICIs), as the safety and activity of these drugs have not been studied on these specific cases, since these patients were excluded from clinical trials leading to approval of ICIs. It is therefore important to gain as much information as possible from the analysis of real-life data, to derive an indication to be adopted in everyday clinical setting. Moreover, therapeutic alternatives other than ICIs are scarce, mainly consisting in chemotherapy and anti-EGFR agents, whose activity is lower than immunotherapy and whose toxicity (particularly with chemotherapy) are not sustainable by this frail population. Here, we describe the current evidence of treatment with ICIs in special populations and conclude that it is necessary to find a balance between treatment risks (toxicities) and benefits (efficacy), as well as engaging a multidisciplinary team of experts to thoroughly manage and treat these patients.

## Introduction

Cutaneous squamous cell carcinoma (cSCC) is the second most common non-melanoma skin cancer, accounting for 20% of skin cancers [1]. Incidence rates change according to skin phenotype, gender (highest risk for men compared with women), and geographic areas: in North America, the incidence changes between Canada (60/100,000 inhabitants) and Arizona (USA) (290/100,000 inhabitants); in Europe, age-standardized incidence is 9–96/100,000 inhabitants for males and 5–68/100,000 inhabitants for females; and in Australia, the incidence reported is 387/100,000 inhabitants [2]. Mortality rates for cSCC are not always well documented; however, 5-year survival rate is estimated to be ranging from 88% for localized disease, to 50% for metastatic disease [3,4]. Most cSCCs are diagnosed in the early stage and are eligible for curative treatment (more than 90% of cases), however, up to 5% of cases may present with a non-resectable disease [5] and other cases may not be eligible for surgery due to comorbidities. Immune checkpoint inhibitors (ICIs) offer new therapeutic perspectives to cSCC not amenable to locoregional treatments, achieving response rates in up to 50% of cases and providing benefits to a subgroup with durable disease control [6]. Despite general good tolerability, immunotherapy could be rarely associated with severe immune-related adverse events (irAEs), due to uncontrolled activation of the immune system. In this regard, immunotherapy safety profile should also be studied in “special populations”, ie, the patients whose comorbidities or frailties excluded them from clinical trials that led to the approval of ICIs. Special populations include patients with dysregulated immune activation (pre-existing autoimmune diseases or immunosuppression due to hematopoietic/solid organ transplant recipients), patients with a compromised immune function (long-term immunosuppression), patients with chronic viral infections, or those affected by lymphoproliferative diseases in which the safety of ICIs is not well studied.

### “Special Population” in Immunotherapy Treatment

Patients receiving solid organ transplantation (SOT) or hematopoietic stem cell transplant (HSCT) require modulation of the immune system to maintain allograft tolerance and avoid rejection, and graft versus host disease (GVHD). Preclinical data showed that the PD-1/PD-L1 axis has an important role in maintaining tolerance, even if not well understood. PD-1/PD-L1 axis is required for the maintenance of T-cell tolerance to prevent alloimmunity following HSCT, and reduces the risk of GVHD [7]. As the intervention with ICIs migh induce alteration in the mechanism of T-cell exhaustion, thus reinvigorating immune response, ICIs could be also responsible for organ rejection [8,9]; as a consequence SOT recipients were usually excluded from main ICI clinical trials. Data in literature regarding the use of ICIs in this population are related to case series and case reports. Rejection rates in patients receiving ICIs are reported in 36% and 54% of liver and kidney transplantation, respectively [10]. Recently, 19 patients receiving liver transplant and 29 patients receiving kidney transplant were treated with ICIs, showing a disease control rate of 35% and an organ rejection rate of 37% and 45% for liver and kidney transplantation, respectively [11]. A literature search and review of 27 articles reported a 40% rate of allograft rejection in renal transplant recipients treated with ICIs for advanced solid cancers (mainly melanoma); 17% of these patients achieved a partial response to immunotherapy [12]. A large systematic review on SOT recipients undergoing ICIs due to advanced solid cancer confirmed the rejection rate of approximately 40%, similar across the type of ICIs and primary tumor histology [13]. An important issue is how to reduce risk of organ rejection without affecting immunotherapy efficacy: lowering or withdrawing immunosuppressive regimens is possible, however this needs to be discussed in multidisciplinary tumor board [14,15]. Intriguingly, immune-suppressive agents used in combination with corticosteroids could modulate the risk of organ rejection. It seems that the use of mTOR inhibitors, instead of calcineurin inhibitors, could be an option for SOT recipients with cancer requiring ICI treatment, due to the reduction of the risk to develop organ rejection, combined with the known antitumor activity [16,17].

ICIs are currently used in hematological diseases as salvage therapies in patients affected by Hodgkin lymphoma, while there is limited evidence of their use in other types of lymphoma and lymphoproliferative disease [18]. Patients who need ICIs treatment for solid cancer with a history of hematological disease could have previously received an immunotherapy treatment or could have also received an autogenic/allogenic transplant. If treatment with ICIs was well tolerated by hematological patients, further retreatment with ICIs is not contraindicated [8]. On the other hand, patients who previously received an allogeneic transplant due to hematological diseases need to be carefully evaluated: ICIs treatment increases the risk of GVHD; intriguingly anti-CTLA-4 ipilimumab appears to have higher safety in this setting [19–21]. Well-known risk factors that increase the risk of GVHD during ICIs treatment are: a previous GVHD, the status of chronic GVHD, and previous toxicities [8].

Another group of patients at higher risk of toxicities by immunotherapy are those suffering from autoimmune diseases. In these cases, further immune stimulation could lead to a possible flare in immune activation, with clinical consequences. Therefore, patients affected by autoimmune diseases, even if silent, are usually excluded from clinical trials with ICIs, for fear of new, potentially life-threatening, symptoms’ appearance. Preclinical data showed that mice deficient for CTLA-4 or PD-1/PD-L1 may develop serious immune-mediated symptoms, including one death due to fulminant autoimmune disease [22]. However, cancer patients suffering from previous autoimmune disease do not represent a small portion of cancer patients: for instance, it is estimated that 13% of lung cancer patients had a positive anamnesis for autoimmune disease [23]. A series of 30 patients affected by melanoma, treated with ICIs and with concurrent diagnosis of autoimmune disease showed that 27% of patients had exacerbations of their autoimmune disease, 33% developed immune-related adverse events (irAEs) requiring treatment, and one patient with psoriasis died of autoimmune colitis; the response rate was comparable with other ipilimumab clinical trials [24]. Safety and efficacy of anti-PD-1/anti-PD-L1 therapy in cancer patients affected by autoimmune disorders have been shown in various series: rates of irAEs and autoimmune flare were consistent, ranging from 23% to 44%, and efficacy did not differ from clinical trials in patients without autoimmune disorders. To note, irAEs and autoimmune flares responded well to a classic therapeutic algorithm [25–28]. Is important to consider cancer patients with specific circulating autoimmune markers or antibodies without evidence of autoimmune disease: this population experienced greater efficacy and greater toxicities from ICIs treatment [29]. Recently, an Italian series showed no difference in grade 3–4 irAEs among 751 cancer patients treated with anti-PD-1 agents comparing patients with or without autoimmune disorders [30].

Chronic immune suppression due to treatment of the aforementioned conditions may hind the effects of immunotherapy by blocking T-cell activity. Patients receiving chronic immunosuppressant agents or high-dose steroids are usually excluded from trials with ICIs, consequently, data in literature is limited. Experiences from ipilimumab trials, given the frequency of primary or secondary adrenal insufficiency, showed that a physiologic (replacement) dose of corticosteroids did not influence ICIs activity [31,32]. On the other hand, patients with prior autoimmunity receiving high-dose steroids showed less activity of ICIs if compared with patients who did not need high-dose corticosteroids (response rate: 15% vs 40%, respectively) [25]. A study of ipilimumab in patients affected by metastatic melanoma with brain metastasis, confirmed a lower response rate in patients receiving high-dose steroids [33]. Recently, a single-institution study showed that the early start of steroids during immunotherapy could be linked to a lower probability of response and lower survival [34].

### Importance of Considering “Special Populations” with cSCC

The immune suppressed population has an incidence of cSCC that is 65–250-times higher compared with the general population. This draws attention to the central role played by the immune system in the development of cSCC [35]. Immunosuppression could be iatrogenic, usually due to therapies used for allogenic organ transplants, hematological or autoimmune diseases, related to HIV, or primary immunodeficiency.

The immune-suppressed population represents a challenging target in the treatment of advanced or metastatic cSCC not amenable to locoregional treatment, as they present with a more aggressive disease and with a higher risk of developing immune related toxicities due to ICIs. cSCC grows more rapidly in immunosuppressed patients and has a higher tendency to develop local and distance recurrences [36]. Moreover, mortality rates are higher for cSCC patients with a history of SOT [37].

Immunotherapy with ICIs anti-PD-1 (cemiplimab and pembrolizumab), are now recommended as first-line treatment for patients with advanced cSCC who are not eligible for surgery or radiotherapy based on results of phase II clinical trials [6,38,39]. However, patients affected by autoimmune disease requiring immunosuppressive agents, anamnesis of prior solid organ transplant, active viral infection requiring specific therapy, such as HIV or hepatitis B virus or hepatitis C virus, and affected by chronic lymphocytic leukemia were excluded by registration drug clinical trials. In addition, international guidelines do not give specific guidance to treat these patiens. Therapeutic alternatives for those populations who are excluded by clinical trials with ICIs are scarce, possibly limited by less activity and more toxicity, mainly based on platinum-based chemotherapy and anti-EGFR-targeted treatment.

Platinum-based chemotherapy for advanced cSCC showed an objective response rate of 44%, median progression free survival (mPFS) and median overall survival (mOS) was 5.5 months and 10.9 months, respectively, and the 3-year survival was 22% [40]. Cetuximab (anti-EGFR-targeted treatment), when employed as monotherapy, showed 27.7% of response rate with 2 complete responses out of 36 patients, responses were rapid, and 61% of patients had serious adverse events (grade 3–4) [41].

Within this scenario it becomes strikingly important to balance risks (toxicities) and benefits (efficacy) of ICIs, when managing treatment for the “special population” excluded from ICIs in previous clinical trials.

### Immunotherapy in cSCC and Special Patient Populations

There is a lack of prospective data in the literature regarding immunotherapy in cSCC “special populations”. Data are mostly derived from case reports, case series, or retrospective data. An immune-suppressed patient with HIV and metastatic cSCC has been reported to experience stabilization of disease and no side effects with pembrolizumab as third-line systemic treatment [42].

A patient with a kidney transplant showed complete pathological response to the combination of nivolumab + ipilimumab, but experienced allograft rejection [43]. Two other cases were affected by advanced scalp squamous cell carcinoma and kidney transplant recipients have been successfully treated with cemiplimab [44,45]. Real-world retrospective series on patients affected by cSCC [46] showed a response rate of 42% and duration of response of 2 years (range: 1–32 months) with cemiplimab in immunosuppressed patients, described as patients who needs chronic immune suppression due to HIV, hematologic malignancies, SOT recipients, and autoimmune disorders. Out of 5 SOT recipients, the duration of response was 20 months and just 1 patient experienced acute allograft rejection. A low rate of rejection was probably due to the fact that 3 out 5 SOT recipients were receiving immunosuppression with prednisone + mTOR inhibitors. Intriguingly, the rate of grade 3 or 4 adverse events (21%) was not statistically different between the immunosuppressed population and the immunocompetent population. All patients with rheumatologic disease experienced lower-grade immune exacerbation. A French case series on patients receiving ICIs for advanced cSCC [47] showed 3 serious adverse events out of 8 immune-compromised patients (due to hematological disease and HIV infection). The CemiplimAb-rwlc Survivorship and Epidemiology (C.A.S.E.) study, is a prospective study aimed at evaluating safety and effectiveness of cemiplimab in a real-life setting. Among the 138 patients enrolled up to now, 30 were immunocompromised or immunosuppressed; 1 patient experienced an acute renal failure, and no treatment-related deaths were reported, while the overall response rate (45.5%) was super-imposable of that obtained in other clinical trials with no “special populations” [48].

## Conclusion

### What to do Before Starting ICIs in a cSCC Special Patient Population?

Management of patients with advanced cSCC changed with the introduction of ICIs; however, treatment for the “special population” remains an important unmet medical need. It is important to carefully detail immunotherapy’s pros and cons to patients, considering the impact on prognosis and the possible toxicities that could develop and could potentially be life-threatening. To reduce the risk of possible immune complications, in the case of SOT recipients, the change of the immunosuppressive regimens from the inhibitor of calcineurin to mTOR inhibitors is indicated. Steroids are often employed as preventative measures in high-risk cases, even if there are no clear dose and time indications. We suggest adjusting the dose of steroids according to the foreseen risk and the developed toxicities, to balance the need of limiting adverse effects of exaggerated immune activation with the possibility to achieve clinical response and not to compromise therapeutic effectiveness. Each case of cSCC patient belonging to the “special population” needs to be discussed and treated within a multidisciplinary team of experts, aiming to offer the best possible therapeutic armamentarium built *ad hoc* depending on the required needs.

Lastly, considering constraints of enrolling these patients in randomized clinical trials, the enrollment of “special patient populations” treated with immunotherapy in observational studies may contribute to increase the understanding of their treatment opportunities.

## References

[1] Rogers HW, Weinstock MA, Harris AR (2010). Incidence Estimate of Nonmelanoma Skin Cancer in the United States, 2006. Arch Dermatol.

[2] Green AC, Olsen CM (2017). Cutaneous squamous cell carcinoma: an epidemiological review. Br J Dermatol.

[3] Boyers LN, Karimkhani C, Naghavi M, Sherwood D, Margolis DJ, Hay RJ (2014). Global mortality from conditions with skin manifestations. J Am Acad Dermatol.

[4] Eisemann N, Jansen L, Castro FA (2016). Survival with nonmelanoma skin cancer in Germany. Br J Dermatol.

[5] Patel R, Chang ALS (2019). Immune Checkpoint Inhibitors for Treating Advanced Cutaneous Squamous Cell Carcinoma. Am J Clin Dermatol.

[6] Rischin D, Khushalani NI, Schmults CD, Guminski AD, Chang ALS, Lewis KD (2020). Phase II study of cemiplimab in patients (pts) with advanced cutaneous squamous cell carcinoma (CSCC): Longer follow-up. JCO.

[7] Saha A, Aoyama K, Taylor PA (2013). Host programmed death ligand 1 is dominant over programmed death ligand 2 expression in regulating graft-versus-host disease lethality. Blood.

[8] Johnson DB, Sullivan RJ, Menzies AM (2017). Immune checkpoint inhibitors in challenging populations: Immune Therapy in Difficult Populations. Cancer.

[9] Chae YK, Galvez C, Anker JF, Iams WT, Bhave M (2018). Cancer immunotherapy in a neglected population: The current use and future of T-cell-mediated checkpoint inhibitors in organ transplant patients. Cancer Treatment Reviews.

[10] Gassmann D, Weiler S, Mertens JC, Reiner CS, Vrugt B, Nägeli M (2018). Liver Allograft Failure After Nivolumab Treatment—A Case Report With Systematic Literature Research. Transplantation Direct.

[11] De Bruyn P, Van Gestel D, Ost P, Kruse V, Brochez L, Van Vlierberghe H (2019). Immune checkpoint blockade for organ transplant patients with advanced cancer: how far can we go?. Current Opinion in Oncology.

[12] Manohar S, Thongprayoon C, Cheungpasitporn W, Markovic SN, Herrmann SM (2020). Systematic Review of the Safety of Immune Checkpoint Inhibitors Among Kidney Transplant Patients. Kidney International Reports.

[13] d’Izarny-Gargas T, Durrbach A, Zaidan M (2020). Efficacy and tolerance of immune checkpoint inhibitors in transplant patients with cancer: A systematic review. Am J Transplant.

[14] Esfahani K, Al-Aubodah T-A, Thebault P, Lapointe R, Hudson M, Johnson NA (2019). Targeting the mTOR pathway uncouples the efficacy and toxicity of PD-1 blockade in renal transplantation. Nat Commun.

[15] Rzeniewicz K, Larkin J, Menzies AM, Turajlic S (2021). Immunotherapy use outside clinical trial populations: never say never?. Annals of Oncology.

[16] Luan FL, Hojo M, Maluccio M, Yamaji K, Suthanthiran M (2002). Rapamycin blocks tumor progression: unlinking immunosuppression from antitumor efficacy1. Transplantation.

[17] Karia PS, Azzi JR, Heher EC, Hills VM, Schmults CD (2016). Association of Sirolimus Use With Risk for Skin Cancer in a Mixed-Organ Cohort of Solid-Organ Transplant Recipients With a History of Cancer. JAMA Dermatol.

[18] Delanoy N, Michot J-M, Comont T, Kramkimel N, Lazarovici J, Dupont R (2019). Haematological immune-related adverse events induced by anti-PD-1 or anti-PD-L1 immunotherapy: a descriptive observational study. The Lancet Haematology.

[19] Haverkos BM, Abbott D, Hamadani M, Armand P, Flowers ME, Merryman R (2017). PD-1 blockade for relapsed lymphoma post–allogeneic hematopoietic cell transplant: high response rate but frequent GVHD. Blood.

[20] Davids MS, Kim HT, Bachireddy P, Costello C, Liguori R, Savell A (2016). Ipilimumab for Patients with Relapse after Allogeneic Transplantation. N Engl J Med.

[21] Ijaz A, Khan AY, Malik SU, Faridi W, Fraz MA, Usman M (2019). Significant Risk of Graft-versus-Host Disease with Exposure to Checkpoint Inhibitors before and after Allogeneic Transplantation. Biology of Blood and Marrow Transplantation.

[22] Klocke K, Sakaguchi S, Holmdahl R, Wing K (2016). Induction of autoimmune disease by deletion of CTLA-4 in mice in adulthood. Proc Natl Acad Sci USA.

[23] Khan SA, Pruitt SL, Xuan L, Gerber DE (2016). Prevalence of Autoimmune Disease Among Patients With Lung Cancer: Implications for Immunotherapy Treatment Options. JAMA Oncol.

[24] Johnson DB, Sullivan RJ, Ott PA (2016). Ipilimumab Therapy in Patients With Advanced Melanoma and Preexisting Autoimmune Disorders. JAMA Oncology.

[25] Menzies AM, Johnson DB, Ramanujam S (2017). Anti-PD-1 therapy in patients with advanced melanoma and preexisting autoimmune disorders or major toxicity with ipilimumab. Annals of Oncology.

[26] Weinstock C, Singh H, Maher VE, Kim G, Pazdur R (2017). FDA analysis of patients with baseline autoimmune diseases treated with PD-1/PD-L1 immunotherapy agents. JCO.

[27] Danlos F-X, Voisin A-L, Dyevre V (2018). Safety and efficacy of anti-programmed death 1 antibodies in patients with cancer and pre-existing autoimmune or inflammatory disease. European Journal of Cancer.

[28] Leonardi GC, Gainor JF, Altan M (2018). Safety of Programmed Death–1 Pathway Inhibitors Among Patients With Non–Small-Cell Lung Cancer and Preexisting Autoimmune Disorders. JCO.

[29] Toi Y, Sugawara S, Kawashima Y (2018). Association of Immune-Related Adverse Events with Clinical Benefit in Patients with Advanced Non-Small-Cell Lung Cancer Treated with Nivolumab. The Oncol.

[30] Cortellini A, Buti S, Santini D (2019). Clinical Outcomes of Patients with Advanced Cancer and Pre-Existing Autoimmune Diseases Treated with Anti-Programmed Death-1 Immunotherapy: A Real-World Transverse Study. The Oncol.

[31] Ribas A, Puzanov I, Dummer R, Schadendorf D, Hamid O, Robert C (2015). Pembrolizumab versus investigator-choice chemotherapy for ipilimumab-refractory melanoma (KEYNOTE-002): a randomised, controlled, phase 2 trial. The Lancet Oncology.

[32] Weber JS, D’Angelo SP, Minor D, Hodi FS, Gutzmer R, Neyns B (2015). Nivolumab versus chemotherapy in patients with advanced melanoma who progressed after anti-CTLA-4 treatment (CheckMate 037): a randomised, controlled, open-label, phase 3 trial. The Lancet Oncology.

[33] Margolin K, Ernstoff MS, Hamid O (2012). Ipilimumab in patients with melanoma and brain metastases: an open-label, phase 2 trial. The Lancet Oncology.

[34] Maslov DV, Tawagi K, Kc M, Simenson V (2021). Timing of steroid initiation and response rates to immune checkpoint inhibitors in metastatic cancer. J Immunother Cancer.

[35] Sylvie E, Jean K, Alain C (2003). Skin Cancers after Organ Transplantation. The New England Journal of Medicine.

[36] Que SKT, Zwald FO, Schmults CD (2018). Cutaneous squamous cell carcinoma. J Am Acad Dermatol.

[37] Garrett GL, Lowenstein SE, Singer JP, He SY, Arron ST (2016). Trends of skin cancer mortality after transplantation in the United States: 1987 to 2013. J Am Acad Dermatol.

[38] Hughes BGM, Munoz-Couselo E, Mortier L (2021). Pembrolizumab for locally advanced and recurrent/metastatic cutaneous squamous cell carcinoma (KEYNOTE-629 study): an open-label, nonrandomized, multicenter, phase II trial. Annals of Oncology.

[39] https://www.ema.europa.eu/en/documents/product-information/entyvio-epar-product-information_it.pdf.

[40] Jarkowski A, Hare R, Loud P (2016). Systemic Therapy in Advanced Cutaneous Squamous Cell Carcinoma (CSCC): The Roswell Park Experience and a Review of the Literature. American Journal of Clinical Oncology.

[41] Maubec E, Petrow P, Scheer-Senyarich I, Duvillard P, Lacroix L, Gelly J (2011). Phase II Study of Cetuximab As First-Line Single-Drug Therapy in Patients With Unresectable Squamous Cell Carcinoma of the Skin. JCO.

[42] Borradori L, Sutton B, Shayesteh P, Daniels GA (2016). Rescue therapy with anti-programmed cell death protein 1 inhibitors of advanced cutaneous squamous cell carcinoma and basosquamous carcinoma: preliminary experience in five cases. Br J Dermatol.

[43] Miller DM, Faulkner-Jones BE, Stone JR, Drews RE (2017). Complete pathologic response of metastatic cutaneous squamous cell carcinoma and allograft rejection after treatment with combination immune checkpoint blockade. JAAD Case Reports.

[44] Paoluzzi L, Ow TJ (2021). Safe Administration of Cemiplimab to a Kidney Transplant Patient with Locally Advanced Squamous Cell Carcinoma of the Scalp. Current Oncology.

[45] Ali SA, Arman HE, Patel AA, Birhiray RE (2020). Successful Administration of Cemiplimab to a Patient With Advanced Cutaneous Squamous Cell Carcinoma After Renal Transplantation. JCO Oncology Practice.

[46] Hanna GJ, Ruiz ES, LeBoeuf NR (2020). Real-world outcomes treating patients with advanced cutaneous squamous cell carcinoma with immune checkpoint inhibitors (CPI). Br J Cancer.

[47] Valentin J, Gérard E, Ferte T, Prey S, Dousset L, Dutriaux C (2021). Real world safety outcomes using cemiplimab for cutaneous squamous cell carcinoma. Journal of Geriatric Oncology.

[48] Rabinowits G, Park SJ, Ellison DM (2021). Checkpoint inhibition in immunosuppressed or immunocompromised patients with advanced cutaneous squamous cell carcinoma (CSCC): Data from prospective CemiplimAb-rwlc Survivorship and Epidemiology (C.A.S.E.) study. JCO.

